# Safety Aspects of Genetically Modified Lactic Acid Bacteria

**DOI:** 10.3390/microorganisms8020297

**Published:** 2020-02-21

**Authors:** Tina Vida Plavec, Aleš Berlec

**Affiliations:** 1 Department of Biotechnology, Jožef Stefan Institute, SI-1000 Ljubljana, Slovenia; tina.plavec@ijs.si; 2 Faculty of Pharmacy, University of Ljubljana, SI-1000 Ljubljana, Slovenia

**Keywords:** lactic acid bacteria, generally recognized as safe, engineering, safety, cell factory

## Abstract

Lactic acid bacteria (LAB) have a long history of use in the food industry. Some species are part of the normal human microbiota and have beneficial properties for human health. Their long-standing use and considerable biotechnological potential have led to the development of various systems for their engineering. Together with novel approaches such as CRISPR-Cas, the established systems for engineering now allow significant improvements to LAB strains. Nevertheless, genetically modified LAB (GM-LAB) still encounter disapproval and are under extensive regulatory requirements. This review presents data on the prospects for LAB to obtain ‘generally recognized as safe’ (GRAS) status. Genetic modification of LAB is discussed, together with problems that can arise from their engineering, including their dissemination into the environment and the spread of antibiotic resistance markers. Possible solutions that would allow the use of GM-LAB are described, such as biocontainment, alternative selection markers, and use of homologous DNA. The use of GM-LAB as cell factories in closed systems that prevent their environmental release is the least problematic aspect, and this is also discussed.

## 1. Introduction

Lactic acid bacteria (LAB) are a heterogeneous group of Gram-positive bacteria that are comprised of the genera *Lactobacillus*, *Lactococcus*, *Streptococcus*, *Enterococcus*, *Leuconostoc*, *Carnobacterium*, *Oenococcus*, *Pediococcus*, *Tetragenococcus*, *Vagococcus*, and *Weissella* [1]. Due to their historical long-term use they are regarded as safe, and some of the species are used as probiotics, as they have beneficial effects on people. Moreover, when describing their characteristics, LAB are often claimed to have a ‘generally recognized as safe’ (GRAS) status, which confirms their safety. 

However, while several LAB species are harmless to humans, from a regulatory point of view, not all LAB can be described as GRAS. The US Food and Drug Administration (FDA) is responsible for GRAS status approval, and thus requires to assess the safety of a specific strain before granting it GRAS status. In this review, GRAS labeling and the procedures to obtain this status are described. In addition, a table with FDA approved LAB strains and their intended use is provided. 

To date, only nongenetically modified (nonGM)-LAB strains have been granted FDA approval. However, genetic engineering of LAB offers various tools to improve the strains and to enable greater viability and stability, and production and growth rates. The possibilities to effectively express therapeutic proteins and to use LAB as vaccines further strengthens their potential uses [2,3]. However, no matter how effective a specific GM-LAB might be, a drawback that stands in the way of its marketing is that it has been genetically manipulated; this is mainly due to low consumer acceptance of GM microorganisms (particularly in the European Union [EU]) and regulatory restrictions as to their use. At the combined 2018 International Probiotics Association World Congress and Probiota, it was noted that GM probiotics would be perfect from a scientific point of view, and would probably not encounter problems with their registration with the European Food Safety Authority (EFSA). However, the indifference of consumers and industry to GM organisms (GMOs) prevents their acceptance, and accordingly, only naturally occurring strains obtain approval (adapted from [4]). 

New methods such as recombineering and ‘clustered regularly interspaced short palindromic repeats’ (CRISPR)-Cas9 now allow highly controlled implementation of precise modifications [5]. While certain levels of acceptance of these methods has been achieved in the USA, they have not been differentiated from other methods of genetic modification in Europe. The obstacles that can be encountered when trying to bring genetically engineered LAB to the market are discussed here, as well as the steps that would be required for them to reach better acceptance.

## 2. ‘Generally Recognized as Safe’ Status: Definition and Determination

The GRAS status indicates that when a substance is added to food, it is considered safe by the FDA. The FDA can categorize microorganisms and their derivatives either as food additives that are approved for specific uses, or as GRAS substances. Since 1997, a substance has been considered as GRAS if: (a) it was used in food before 1958 (i.e., GRAS status is based on a history of safe use in food, with the requirement for a substantial history of consumption in food by a significant number of consumers); or (b) scientific procedures have been undertaken that require the same quantity and quality of evidence as would be required to satisfy food-additive regulations. A GRAS substance and a food additive are distinguished according to who performs the evaluation. For a food additive, the FDA determines the safety of the ingredient, whereas for a GRAS substance, the determination can be made by qualified experts outside of the FDA. 

A full listing of notices and response letters together with a search engine is available on the GRAS notice website (https://www.accessdata.fda.gov/scripts/fdcc/?set=GRASNotices), which allows searches through the inventory that is up-dated monthly. Supplementary Table S1 gives the LAB for which GRAS notices have been submitted to the FDA up to December 2019, and which are intended as live food additives. It only includes LAB for which the FDA has not questioned the GRAS conclusion of the notifier. For the use of harmless LAB, as optional ingredients in specified standardized foods, such as *Lactobacillus acidophilus*, prior approvals have been recognized (i.e., before 1958). Use of these bacteria is permitted in cultured milk (which includes buttermilk), sour cream, cottage cheese, and yogurt, provided that the mandatory cultures of *Lactobacillus bulgaricus* and *Streptococcus thermophillus* are also used in the yogurt.

Whereas GRAS status applies to the regulatory framework in the USA, in Europe, the EFSA uses the Qualified Presumption of Safety (QPS) procedure to evaluate products with microorganisms and to assess the risks associated with human, animal, and environmental use [6]. For the European regulatory framework and the steps required for QPS status, the reader is referred to [7], where a detailed description is provided.

## 3. The Consequence of Genome Modification

Genome modification of LAB can result in either inactivation of a given cell function or introduction of a new function. To achieve the inactivation of a function, a gene can be either deleted or mutated so as to diminish its function. *Lactobacillus johnsonii* has three active bile salt hydrolase (*bsh*) genes and can persist in the mouse gut; *Lactobacillus helveticus*, on the other hand, cannot persist because it has a frame-shift in the *bsh* gene resulting in its inactivation [8]. *L. lactis* NZ3000 carries an in-frame deletion of the chromosomal *lacF* gene, and therefore it will only grow when lactose is provided [9]. Developments in the field have brought new techniques that now allow precise removal or replacement of genetic elements, and that have already been successfully applied to LAB [10,11], often with the aim of achieving biocontainment and avoiding the spread of unwanted genetic elements into the environment. 

When introducing a new function, a gene can be activated by mutation of the promotor (e.g., to allow or increase protein expression) or by alteration of a gene by point mutation (e.g., to increase the catalytic activity of the enzyme) [12,13]. A new function can also be introduced through the stable genome integration of a new gene. Genome integration results in low numbers of gene copies, but the integrant is not dependent on environmental or developmental factors [14]. Another option is to introduce a new gene with a plasmid [10,11,15]; in this case, multiple copies of recombinant genes can be introduced by increasing the plasmid copy number. 

## 4. Acceptable Methods for Genome Modification in the European Union

### 4.1. Natural Methods

Natural methods of genome modification are acceptable if they avoid the use of recombinant DNA. Natural transformation of LAB can occur through conjugation, phage transduction, or natural competence. This last is considered as the main mode of horizontal gene transfer in prokaryotes. Conjugation is the transfer of genetic material between bacterial cells by direct cell-to-cell transfer of DNA. Although conjugative plasmids and transposons are very common in LAB, the underlying mechanisms are not fully understood yet [10,16]. For phage transduction, the DNA transfer is mediated by bacteriophages, which are viruses that can infect bacteria and manipulate the bacterial replication, transcription, and translation machineries to drive their own proliferation. Phages that infect LAB have been thoroughly investigated, and different species of LAB have been screened for susceptibility to specific phages [17,18,19,20,21]. The third pathway of natural competence in prokaryotes is a developmental process in which exogenous DNA is translocated through the native DNA-uptake machinery. The process is genetically encoded, with a number of genes involved in competence regulation. However, the function of these genes and their contributions to the survival and evolution of the host are still not known [22]. The use of natural methods of genome modification is limited due to the number of modifications available. To date, conjugation has been exploited to some extent. The bacteriophage resistance of *L. lactis* CHCC1915 and CHCC1916 was improved by conjugative transfer of the bacteriophage resistance plasmid pCI1750 from *L. lactis* UC653, which harbors the AbiG resistance system [23]. 

### 4.2. Random Mutagenesis

However, to obtain mutant LAB with the desired functions in biotechnological industries, random physical and chemical mutagenesis have been applied most often [24]. Ultraviolet-induced mutagenesis has been tested for rifampicin and streptomycin resistant mutants of *Lb. acidophilus*, *Lactobacillus delbrueckii*, and *L. lactis*. Their intrinsic resistance to ultraviolet irradiation was 1-2 log higher than that of *Escherichia coli* [25]. With *Lactobacillus gasseri*, which is used in the production of meat products and is sensitive to sodium chloride and sodium nitrite, ultraviolet irradiation generated mutants that were stable in the presence of both compounds [26]. The chemical mutagen ethyl methane sulfonate has been mostly applied for random mutagenesis. *Lactobacillus pentosus* subjected to mutagenesis by ethyl methane sulfonate can withstand higher xylose levels and produce larger amounts of lactic acid through conversion of xylose into lactic acid [27]. Ethyl methane sulfonate mutagenesis of *L. lactis* has produced variants with improved riboflavin production [24].

However, there are several drawbacks of such random mutagenesis methods. They are not targeted, and can introduce various random mutations into the genomes of interest, after which the characterization and selection of the subset of target variants is required. In this way, undesired mutations can occur that require further characterization of the isolated strains. Recombinant DNA technology, on the other hand, is targeted and enables precise modifications to be made [23]. 

## 5. Unacceptable Methods for Genome Modification in the European Union

### 5.1. Transformation with Plasmids and Genome Integration

Genetic modifications in LAB can be achieved through either plasmid-encoded expression systems or chromosome modifications. A number of plasmids used for gene cloning, expression, and secretion for LAB have been developed [15]. Plasmid-encoded expression allows for the use of constitutive or inducible promoters, with the latter favored on account of the better control over the recombinant protein expression. The nisin-controlled gene expression system is the most frequently used lactococcal expression system [28], the sakacin system is comparably effective and has been applied to several *Lactobacillus* strains [29,30,31]. 

Chromosome modifications can be achieved through stable genome integration of recombinant genes. Traditional chromosomal integration strategies have included insertion of sequence elements, phage-integration systems, and homologous-recombination-based systems, with homologous recombination as the most frequently used for chromosomal insertions, deletions, and gene replacements [5,11]. Homologous recombination systems are based mainly on the use of nonreplicative suicide vectors, which contain sequences that are homologous to the insertion site. The pSA3-based suicide vector (pTRK327) with the IS1223 insert of *Lb. johnsonii* have been used to promote gene insertion into several *Lactobacillus* species [32]. Furthermore, the development of these LAB genome modification methods has focused on approaches that leave no selectable markers or residual bases. Several systems have been described. A pORI thermosensitive plasmid with temperature-dependent replication has been used for site-specific replacement of chromosomal DNA sequences in *L. lactis*, *Lb. acidophilus*, and *Lb. gasseri* [33,34,35]. The *upp* gene that encodes uracil phosphoribosyltransferase was applied to *L. lactis*, *Lb. acidophilus* and *Lactobacillus casei* as a counter-selectable marker for positive selection of double recombinants [36,37]. A Cre-lox-based system was applied to *Lactobacillus plantarum* for multiple gene deletions and selectable-marker removal [38], and to *L. lactis* to achieve large genome rearrangements [39]. 

More recent genome-engineering tools, such as recombineering and CRISPR-Cas9–based engineering, facilitate faster and more straight-forward development of targeted mutations of the bacterial chromosome. 

### 5.2. Recombineering

Single-strand DNA recombineering is a phage-encoded homologous recombination system that uses short homologous DNA (i.e., 50 bp). Single-stranded oligonucleotides with the desired mutation are used as the substrates, and protein β from λ phage, or RecT recombinase from the Rac prophage, are used to mediate incorporation of an oligonucleotide into the genome [5]. Recombineering has been demonstrated to be an effective tool in the incorporation of chromosomal mutations in *Lactobacillus reuteri* and *L. lactis* [40]. Moreover, the efficiency of recombineering in genomic engineering of *L. lactis* [41] and *L. reuteri* was improved by the use of CRISPR-Cas9 [42], which is addressed below in more detail. 

### 5.3. CRISPR-Cas9–Supported Genome Rearrangement, Recombineering, and Gene Integration

CRISPR-Cas systems have adaptive immune functions in prokaryotic organisms and they are used as programmable genome editing tools for precision genome engineering of eukaryotic and bacterial cells [10,43]. Cas9 is a DNA endonuclease of type II CRISPR-Cas systems, and is the most used of the Cas proteins. Cas9 uses a guide-RNA to form base pairs with the target DNA sequence, which is then followed by cleavage of this DNA and introduction of a site-specific double-strand break [44]. Its application to DNA targeting of four genomic islands in *Streptococcus thermophilus* resulted in generation of stable mutants that collectively lacked a total of 7% of their genome [45]. Cas9 has also been used for removal of plasmids, integrative conjugative elements, and prophages in *L. lactis*, where specific genetic loci were targeted using the pNZCRISPR and pLABTarget vectors [46,47]. Alternative Cas9 endonucleases can further improve the genetic engineering of bacterial strains. Replacement of wild-type Cas9 with the Cas9D10A (i.e., nickase), a variant of Cas9 that makes single-stranded nicks instead of double-stranded breaks due to a mutation in one of the two active sites of Cas9, decreased Cas9-related lethality and increased the efficiency of genome engineering in *Lb. casei* [48]. Another mutant of Cas9, nuclease-inactivated Cas9 (i.e., catalytically dead Cas9), enables CRISPR interference, which results in precise, targeted gene silencing. CRISPR interference is achieved by specific single-guide-RNA-guided binding of nuclease-inactivated Cas9 to target genes and promoters that leads to obstruction of RNA polymerase [49]. Proof of principle of CRISPR interference activity in *L. lactis* was demonstrated by silencing the *upp* gene [47]. For some species, Cas9 is toxic and cannot be used for genetic engineering [10]; therefore, alternative Cas proteins have been developed, such as the Cas9 variant ThermoCas9 [50], Cpf1 and C2c1/2/3 [51], and Cas12a, with this last combined with single-stranded DNA recombineering to improve precision [52,53,54]. Another improvement to the field is base editing, where a fusion protein that contains a catalytically impaired Cas9 variant coupled to cytidine deaminase allows C to T (or G to A) substitutions [10,55,56]. Although this base editing has not been applied to LAB yet, it can be used to target specific genes or to introduce stop codons [57,58,59]. These recent techniques should accelerate the progression in the field of LAB genome editing, and consequentially, provide for higher specificity, efficiency, and through-put. 

## 6. The Main Issues with Regard to Genetically Modified Lactic Acid Bacteria, and the Attempts to Address Them

**Figure 1:**
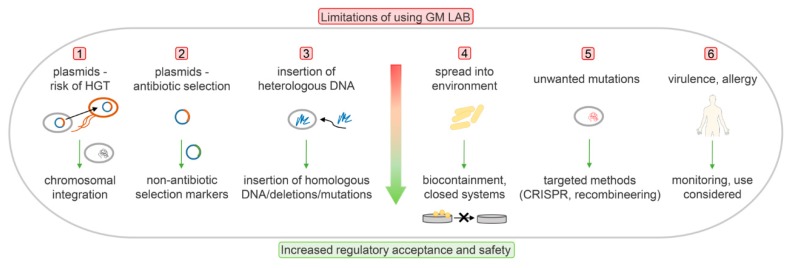
Limitations in the development of genetically modified lactic acid bacteria (GM-LAB), and the ways to overcome the potential problems and to increase the regulatory acceptance. HGT: horizontal gene transfer. CRISPR: clustered regularly interspaced short palindromic repeats.

Genetic modification itself is regarded as problematic from both the regulatory legislative view and the consumer perspective. To date, none of the GM-LAB strains have been brought to the market for therapies or as food supplements. Instead, the industry has focused on avoiding the use of recombinant DNA technology, and has applied spontaneous mutagenesis instead. Challenges in developing GM-LAB and the ways to overcome the potential problems and reach regulatory acceptance are presented schematically in Figure 1.

### 6.1. Biocontainment

Biocontainment is the strategy used to prevent both lateral dissemination of genetic material to other bacteria and environmental dissemination of engineered bacterial strains, with the subsequent accumulation in the environment. According to The National Institutes of Health, a GMO escape rate below 1 in 10^8^ cells is considered to provide acceptable safety [60]. The frequency of any escape of mutants must be low enough to assure that the mutant is unlikely to survive. 

Biocontainment systems can be active or passive. Active containment systems enable killing of the host through activation of a killing gene or repression of an essential gene, the expression of which is tightly controlled by an environmentally responsive element [61]. However, mutations that either inactivate a killing gene or result in constitutive expression of an essential gene can occur. Moreover, in active systems, the amounts of foreign DNA introduced are often larger than in passive systems. Active systems usually depend on a plasmid, which needs to be integrated into the bacterial chromosome. Subsequently, demonstration of LAB functionality is required after plasmid integration into the bacterial chromosome. On the other hand, passive containment systems are robust and very simple in design, and they are based mainly on complementation of an auxotrophy or gene defect by supplementing another gene or essential metabolite that is normally not present in the environment. The major drawback of passive systems is that they are often bacteriostatic rather than bactericidal. However, bactericidal effects can be obtained through the combination of two auxotrophies [61]. 

Bactericidal auxotrophy was achieved in a thymidine synthase (*thyA*) mutant of *L. lactis* by replacing the thymidylate synthase gene *thyA* of *L. lactis* with a synthetic human *IL10* gene. When deprived of thymidine or thymine, the viability of *L. lactis* dropped, and therefore prevented its accumulation in the environment [62]. After its validation in pigs, this was approved by the Dutch authorities as an experimental therapy for humans with inflammatory bowel disease [62]. Furthermore, the *thyA–L. lactis* system has been recognized by the Belgian Biosafety Advisory Council, Health Canada, the Swedish Medical Products Agency, and the Canadian Environmental Protection Agency [63]. Recombinant *Lb. casei ΔthyA* that expresses bovine lactoferricin was also successfully constructed, and only survived in the presence of thymine [64]. A similar auxotroph transgene containment method has targeted the *pyrG* gene, which encodes CTP synthase, and is thus responsible for converting UTP to CTP during *de-novo* pyrimidine synthesis in *L. lactis*. The cytidine auxotrophy in *L. lactis*, however, was bacteriostatic rather than bactericidal [63]. A double mutation in the genes that encode ThyA and PyrG was tested in *L. lactis* to confer double auxotrophy for both thymidine and cytidine. However, while a bactericidal phenotype was observed in the *thyA* mutant, the combination of two mutations did not result in enhancement of the biological containment of the engineered strain, but instead compromised the bactericidal effect [65]. 

New biocontainment strategies use synthetic gene circuits to control cell proliferation in response to environmental conditions, which are detected by allosterically regulated transcription factors [66,67]. To develop synthetic auxotrophs, strains dependent on an exogenous supply of small synthetic molecules for essential gene expression were constructed in *E. coli*. The biocontainment system contained overlapping safeguards: engineered riboregulators for control of the expression of essential genes; and an engineered addiction module based on nucleases that cleave the host genome [68]. Another *E. coli* synthetic auxotroph that is metabolically depended on nonstandard amino acids for survival has been developed [69]. In addition, a strain of *E. coli* that can only survive at high population densities was engineered. The essential gene expression depended on the presence of the quorum-sensing molecule acyl-homoserine lactone; its presence at high enough concentrations could only be achieved in a high-cell-density environment. These bacteria survived only when they were encapsulated in special capsules, due to their high local density. Bacteria that escaped from a capsule were killed due to the decrease in their density [70]. These new biocontainment strategies could be transferred from *E. coli* to LAB. 

### 6.2. Antibiotic Resistance in Lactic Acid Bacteria and its Use in the Selection of Genetically Modified Organisms

Antibiotic resistance involves several mechanisms for direct inactivation of an active antibiotic molecule, as well as for loss of susceptibility to the antibiotic by modification of the target site or reduction of antibiotic uptake. Frequent use of antibiotics can result in resistant bacterial microorganisms that can transfer their resistance machinery to other microorganisms and become a threat to public health and the environment. Therefore, in 2017, the World Health Organisation initiated a campaign to raise awareness of antimicrobial resistance, including antibiotic resistance, as part of a global program [71,72]. 

Lactic acid bacteria might act as reservoirs for antibiotic resistance (AR) genes. Resistance gene transfer is vertical, so it does not present a safety concern in itself. However, external factors can induce changes that favor horizontal transfer of resistance genes to pathogens through the food chain, which represents a major cause of concern for human and animal health. AR genes can be horizontally transferred from one microorganism to another by transduction (e.g., via bacteriophages) or by transformation between microorganisms (e.g., released DNA taken up by another microorganism) [71]. 

Safety studies on LAB have shown that they can easily develop resistance to antibiotics. Most of the available data have been gathered from opportunistic pathogenic enterococci, especially vancomycin-resistant enterococci, which can cause recurrent hospital-acquired infections [73,74]. However, other LAB have also shown resistance, including commensals, probiotics, and food starters from the genera *Lactobacillus*, *Streptococcus*, and *Enterococcus*. Although this resistance is mainly toward vancomycin, it also applies to other antibiotics, such as erythromycin, tetracycline, gentamicin, chloramphenicol, and others [75,76,77,78]. *Enterococcus faecalis* transferred the plasmid DNA to *E. coli* via conjugation [79], and genetic transfer between *L. lactis* as gene donor and enterobacteria has also been reported [80]. Additionally, transfer of vancomycin resistance from enterococci to commensal *Lb. acidophilus* in vitro and in vivo in the gut of mice has been reported [81]. For the risk of AR gene transfer in the human digestive tract after the intake of probiotics, the addition of probiotic lactobacilli to immuno-compromised patients should be considered. 

Methods for evaluation of antibiotic susceptibility are based on phenotypic detection of antibiotic resistance by measurement of bacterial growth in the presence of the antibiotic under consideration, and molecular identification of any resistant genotypes through polymerase chain reaction (PCR). In LAB, phenotypic susceptibility to antibiotics has to be evaluated via determination of the minimum inhibitory concentrations (MICs) of the most commonly used antibiotics. Most LAB can be evaluated by the method described in ISO 10932: 2010 [82]. Strains of *Enterococcus* should be evaluated using the methods described by the Clinical and Laboratory Standards Institute [76]. PCR-based techniques or micro-arrays can be used as complementary techniques to achieve consistency among evaluations across different laboratory settings [83]. Metagenomic studies use either sequence-driven or function-driven approaches for analysis, both of which are based on next-generation sequencing techniques. Function-driven approaches are also used in the characterization of antibiotic resistance [84]. Here, metagenomic libraries are screened for traits of interest by studying their expression in transformed clones and subjecting them to sequencing and biochemical analysis [85]. A bacterium is considered safe when the MIC is lower than the cut-off level. If the MIC is above the cut-off, the bacterium is resistant to the antibiotic, and its resistance should be confirmed by molecular methods, such as PCR [71,86].

Bacteria with intrinsic resistance are considered acceptable for use in food. Intrinsic resistance is specific for bacterial species or genera. It is normally chromosome-encoded and independent of previous antibiotic exposure, and it can arise through different mechanisms, which include impermeability of the outer membrane, efflux pumps with different substrate specificities, and antibiotic-resistance-modifying enzymes [87]. For bacteria with acquired resistance but without intrinsic resistance, it should be demonstrated whether the resistance is in mobile genetic elements, or if the resistance was acquired by mutation of the bacterial chromosome. Only after showing that the resistance is not transferable using genome analysis can the bacteria be accepted for use [7,86].

Antibiotic resistance genes are frequently exploited in genetic engineering for the selection and maintenance of plasmids. Due to the risks of AR explained above, and to the lack of regulatory acceptance, alternative selection options have been considered to conform to the regulatory requirements (e.g., antibiotic-free systems).

### 6.3. Antibiotic-Free Selection Systems

Expression and gene delivery vectors free of AR genes have been developed to improve the safety of a product. According to their mechanisms of action, these systems can be based on auxotrophic, nonantibiotic dominant and complementary markers, post-segregational killing, RNA interference, or de-repression of an essential gene or minicircle [88]. Nonantibiotic dominant selection markers are straightforward and depend upon their presence in the plasmid of interest. Examples of dominant selection markers depend on bacteriocin resistance or heavy metal resistance [89]. A typical representative of the bacteriocins is nisin, which is produced by certain strains of *L. lactis* and is widely used as a safe and natural preservative in the food industry. Nisin has been licensed in a number of countries around the world because of its antimicrobial activity against a broad range of Gram-positive bacteria, including *Staphylococcus aureus* and *Listeria* species. The first food-grade plasmids based on bacteriocin resistance contained the nisin resistance gene [90,91]. Similarly, selection of *L. lactis* transformants was obtained with a plasmid containing the nisin immunity gene *nisI* as a selection marker [92]. The lactacin F immunity protein (lafI) from *Lb. johsonii* can be used as a food-grade marker for genetic engineering of lactobacilli. In *Lb. johnsonii*, disruption of the *lafI* gene resulted in sensitivity to lactacin F. Immunity to lactacin F in *Lb. johnsonii*, *Lb. acidophilus*, *Lactobacillus fermentum*, and *Lb. gasseri* was restored by transforming these strains with a *lafI*-containing plasmid [92]. In *L. lactis*, cadmium resistance (Cdr) and copper resistance (Cur) have been described as heavy-metal-resistance determinants. The vectors thus prepared have been stably expressed in *L. lactis* [93,94] and successfully applied to *S. thermophilus* [95]. Some of these vectors combined resistance to either cadmium or copper and to nisin [94]. Another dominant selection marker was developed on the basis of a *shsp* plasmid from *S. thermophilus* that encodes a protein with homology to small heat-shock proteins. This system does not depend on bacteriocins or heavy metals, and has been transferred into different *S. thermophilus* and *L. lactis* strains. A recombinant plasmid carrying the *shsp* gene enabled selection at 60 °C or pH 3.5, and the transformed strains have shown growth at 52 °C [96].

Nonantibiotic complementary selection marker systems are based on complementation of specific mutations in a chromosomal gene that encodes an essential step in a particular metabolic pathway or that confers certain properties to an organism. An expression vector that carries a complementation is constructed to complement for gene deletion in the host chromosome. Systems based on auxotrophic markers (which are often used to achieve biological containment) in LAB mainly depend on complementation of thymine or cytidine [62,64,65]. Several systems have been applied to *L. lactis*. *L. lactis* that carried a vector with amber suppressor *supD* as the selectable marker resulted in overexpression of the *pepN* gene [97]. Furthermore, both *L. lactis* and *Lb. plantarum* were made auxotrophic for D-alanine by deletion of an internal fragment of the *alr* gene, which encodes an alanine racemase that catalyzes the interconversion of D-alanine and L-alanine. A plasmid that carried a heterologous *alr* was constructed to complement D-alanine auxotrophy [98]. Sugar utilization markers in LAB are mainly limited to sugars that LAB cannot ferment. These are mostly lactose, melibiose, sucrose, and xylose. The selection principle involves the use of a growth medium that contains a single sugar as a carbon source. Lactose fermentation has been used to develop homologous selection markers based on lactose complementation, where the *lacF* gene that encodes the soluble carrier enzyme IIALac was used as a selection marker in combination with an *L. lactis* strain with an in-frame deletion of the chromosomal *lacF* gene [9]. For *Lb. casei*, a stable lactose-deficient mutant was constructed by inactivation of the phospho-β-galactosidase gene *lacG*. Restored growth on lactose was seen after complementation with a food-grade plasmid that carried the *lacG* gene [99]. The α-galactosidase gene (*aga*) of *Lactococcus raffinolactis* confers a melibiose fermentation phenotype, and this was shown to be an efficient food-grade selection marker for *L. lactis*, *Pediococcus acidilactici* [100] and *S. thermophilus* [101]. Similarly, a food-grade expression vector that contained the α-galactosidase gene of *Lb. plantarum* as a selection marker was developed for *L. lactis* [102]. 

Post-segregational killing is provided by plasmids with a toxin and an antitoxin gene pair. Loss of the plasmid depletes the antitoxin in the cell and allows the toxin to act upon it, which results in the killing of the competitive plasmid-free cells. Furthermore, toxin–antitoxin systems have been proposed to prevent horizontal gene transfer [103,104]. An antitoxin gene would be incorporated into a plasmid, and the loss of the plasmid would result in cell death. Toxin–antitoxin systems have been developed for both Gram-negative [105,106] and Gram-positive [107,108] bacteria. RNA interference systems can also be used as alternative selection markers. These use RNA that can interfere with its complementary sequence, which can thus inhibit gene expression and translation. A plasmid was designed with the essential gene *murA*, which is involved in cell wall synthesis, under the control of the tetracycline (*tet*) operator. The *murA* gene was repressed by a genome-encoded *tet* repressor. In the absence of the plasmid, *murA* expression was repressed by tetR, which led to cell death. In bacteria carrying the plasmid, the RNA interference targeted the tetR sequence. As a consequence, tetR was repressed, which resulted in de-repression of *murA* and cell growth [109,110]. Minicircle vectors are circular DNA sequences that are devoid of the plasmid bacterial backbone, and that have been effectively used in *E. coli*. The absence of an antibiotic resistance gene, bacterial replication origin, and the unmethylated immunogenic CpG sequence makes them a safer alternative system to conventional plasmids [111]. Finally, nonantibiotic resistance systems as selection markers also include genes that confer resistance to nonantibiotic compounds. 

Plasmid maintenance and the use of selection markers can be avoided by using a chromosomal gene integration strategy, as described in the previous sections. Chromosomal integration of genes provides stability and reduces the risk of horizontal gene transfer [112].

### 6.4. Use of Homologous DNA

From the regulatory point of view, it would be preferable to include only the DNA from a homologous host (i.e., the cisgenic DNA) or from GRAS organisms in genetic engineering. On the contrary, introduction of DNA from other species results in a transgenic organism, which is strictly regarded as a GMO by the regulatory authorities [113]. A 4.46-kb food-grade cloning vector pUBU was constructed that was composed of three major components from food-approved organisms: the theta-type replicon from *Tetragenococcus halophilus*, the promoter of the L-lactate dehydrogenase (*ldhL*) gene from *Lb. plantarum*, and the lactococcal cadmium resistance (Cdr) determinant as a dominant selection marker. The newly constructed vector was effective for the transformation of several genera of LAB, including *Enterococcus*, *Lactobacillus*, *Lactococcus*, *Leuconostoc*, *Pediococcus*, and *Tetragenococcus*, and was stable in the bacteria without the selection pressure [114]. Alternatively, a shuttle vector with a removable selection marker allows cloning in *E. coli* and protein expression in LAB. The *E. coli* cassette can be easily excised from the selected recombinant plasmid, and the resulting antibiotic-selection-marker-free vector transformed into the final food-grade expression host *L. lactis* NZ3000 with *lacF* gene deletion [115].

Recent progress that has been driven by CRISPR-Cas systems currently allows precise substitutions or deletions of a single base pair at a predetermined site. Sequences that might be considered undesirable for food applications can be precisely excised or substituted with DNA from other strains or species. The regulatory status of cisgenic strains and strains with gene deletions or single base-pair changes introduced depends on the method of gene modification. In the EU, natural methods are regarded as nonGMO, whereas CRISPR and other recombinant methods are regarded as GMO. 

### 6.5. Virulence Factors

Each bioengineered strain needs to be carefully evaluated for virulence factors, i.e., genes that can potentially cause pathogenicity. Such genes should be avoided, as well as genes that encode enzymes known to be involved in the synthesis of toxic or allergenic compounds, or their precursors [116]. The introduction of foreign DNA can result in the synthesis of new substances, as either proteins or metabolic products, which can also have toxic or allergenic effects. For new proteins, the amino-acid sequences have to be compared with the known proteins for potential homology. Their anti-nutrient activities (e.g., as protease inhibitors, lectins), as well as their stabilities to heat, processing, and degradation have to be evaluated in the appropriate representative gastric and intestinal model systems. To date, no definite tests for predictions of allergenic responses in humans have been reported for such newly expressed proteins [117].

### 6.6. Delayed Adverse Effects

The safety of a product and the reaction of an individual to the product depend on its mode of application (e.g., locally, systemically) and the genetic profile of the consumer. Specific sub-populations can be especially sensitive, such as immuno-compromised individuals, infants, and the elderly. Therefore, post-market surveillance of novel foods has to be carried out to avoid potentially serious adverse effects. Effects on the consumer need to be monitored over a long period of time after the release of any such novel food onto the market. Technical issues due to inconsistent intake can represent a drawback, and it might be difficult to monitor the changes over a long time period [117]. As for the monitoring reported for nonGM-LAB [118], close monitoring would be advised also in the case of GM-LAB following their release onto the market, to prevent delayed adverse effects and to ensure safe consumption. 

### 6.7. Surface Display

Heterologous binding of a recombinant protein to the surface of an unmodified bacterium would enable the use of nonGMO and a less complex regulatory procedure [119]; however, confirmation of a lack of recombinant DNA and viable recombinant bacterial cells would be required. Several heterologous protein-display systems have been successfully developed in LAB. The endolysin Lyb5 that was fused to GFP and expressed in *E. coli* was attached to the surface of various LAB, including *L. lactis*, *Lb. casei*, *Lb. brevis*, *Lb. plantarum*, *Lb. fermentum*, *Lb. delbrueckii*, *Lb. helveticus*, and *S. thermophilus* [120]. The S-layer protein SlpB (LcsB) of *Lactobacillus crispatus* fused to GFP was similarly tested for binding to several LAB strains. Binding of the fusion protein to the cells of *Lb. delbrueckii*, *Lb. brevis*, *Lb. helveticus*, *Lb. johnsonii*, *Lb. crispatus*, *Lactobacillus salivarius*, *S. thermophilus*, and *L. lactis* has been demonstrated. The fluorescence of *Lactobacillus* cells was more intense than that of *L. lactis* and *S. thermophiles* [121]. Moreover, ‘designed ankyrin repeat proteins’ (DARPins) fused to cAcmA have shown heterologous binding to the surface of *Lb. acidophilus*, *Lb. delbrueckii* subsp. *bulgaricus*, *Lb. casei*, *Lb. gasseri*, *Lb. gasseri* K7, *Lactobacillus paracasei*, *Lb. plantarum*, *Lb. reuteri*, *Lactobacillus rhamnosus,* and in particular, *Lb. salivarius* [119]. Recently, the principle of heterologous display was demonstrated with the coating of nonrecombinant *L. lactis* with B-domain–cAM12 fusion proteins [122]. An alternative approach for covalent heterologous surface display on *L. lactis* was achieved through formation of isopeptide bonds between the SpyCatcher/SpyTag and SnoopCatcher/SnoopTag protein/peptide pairs. The tagged model protein-B domain was successfully attached to the cell surface of *L. lactis* to display the corresponding catcher protein [123].

## 7. Genetically Modified Lactic Acid Bacteria as Cell Factories

Genetically modified lactic acid bacteria can produce desired compounds as cell factories in closed systems, which reduces the possibility of the unwanted release of microorganisms into the environment and the risk of contamination. Such applications are therefore a lot less problematic from the regulatory point of view. Genetic engineering of LAB has enabled their application to the expression of recombinant proteins. The most common genera used here are *Lactococcus* and *Lactobacillus* [124,125]. Metabolic engineering of LAB allows modification of the existing metabolic pathways to improve the properties of LAB as food fermentation starters [126]. Different metabolic engineering strategies have been used to reroute metabolic pathways for the production of sweeteners, flavors, aromas, exopolysaccharides, and vitamins [127,128,129]. Moreover, through metabolic modification, GM-LAB can produce metabolites with health benefits or high-value biochemicals, with better production yields achieved [128,130]. The main industrial application of LAB is in the fermentative production of lactic acid, which is also the primary product of their carbohydrate metabolism. Lactic acid has a long history of use in food, cosmetics, pharmaceuticals, chemicals, and agriculture. Moreover, the demand for lactic acid is growing on account of its use as a precursor for production of biodegradable and biocompatible polylactic acid, with has commercial value in the fiber, textile, plasticulture, and packaging industries [11,131]. Dependent on the species of *Lactobacillus,* production of L-, D- or LD-lactic acid is possible [132]. The intrinsic production of compounds of interest is preferable, but this can often result in insufficient quantities. Genetic modification offers the possibility to increase the yield of the compound of interest, as well as to enhance the characteristics of the compound (e.g., its purity). To increase the production of optically pure lactic acid, genome shuffling and disruption or deletion of the lactate dehydrogenase *(ldh*) gene has already been applied. Genome shuffling was applied in *Lb. rhamnosus* to improve glucose tolerance and at the same time to enhance L-lactic acid production [133]. Disruption of D-lactate dehydrogenase (*ldhD*) gene or L-lactate dehydrogenase gene (*ldhL*) resulted in the formation of optically pure L- and D-lactic acids, respectively, by *Pediococcus acidilactici* [134] and D-lactic acid by *Lb. plantarum* [135,136]. *ldhD* gene-deficient *Lb. paracasei* [137] and *Lb. helveticus* [138] have been used to produce L-lactic acid. 

As well as the production of lactic acid, LAB have roles in industrial production of nonfood products (e.g., ethanol production) and functional ingredients, such as vitamins, low-calorie sweeteners, exopolysaccharides, and antimicrobial agents. Metabolically engineered *L. lactis* has been used for industrial bioconversion of ethanol from dairy and corn milling waste [139]. LAB-derived bacteriocins are suitable as food preservatives, such as nisin from *L. lactis,* pediocin from *Pediococcus* strains, and enterocin from *En. faecalis* [140]. Nisin is well known for antibacterial effects against *Listeria* and *Clostridium* spores. It has also been approved as a food additive (E234) in the EU, according to Directive 95/2/EC (EC, 1995) [141]. Nisin production in *L. lactis* has been enhanced [142] and its bactericidal effects increased through genetic engineering of the strain [143]. 

Lactic acid bacteria can be used for the production of nutraceuticals, such as polyols (e.g., sugar alcohols) and vitamins, particularly for the B vitamins, such as riboflavin and folate. Significant overexpression of riboflavin has been achieved through genetic engineering of *L. lactis* [24,144,145]. To increase folate levels, metabolic engineering of *L. lactis* [146], *Lb. gasseri* [147], and *Lb. reuteri* has been performed [148]. For the production of polyols, sorbitol levels were increased by engineering *Lb. casei* and *Lb. plantarum* [149,150]. On the other hand, the use of *ldh*-deficient *L. lactis* increased its production of mannitol [151], although due to the genetic engineering applied, this prevented its GRAS labeling [151,152]. 

Recombinant amylolytic and (hemi-)cellulolytic LAB have been constructed for hydrolysis of polysaccharides, which is otherwise achieved by either physicochemical strategies or enzymatic treatments, both of which have the drawback of co-production of toxic compounds and high cost [153]. Several successful constructions of recombinant amylolytic LAB have been reported, such as secretion of heterologous α-amylase in *Lb. casei* [154], *Lb. plantarum* [136,155], and *L. lactis* [156]. Enzymatic systems for lignocellulose hydrolysis include heterologous (hemi-)cellulase expression, or other enzyme expression (e.g., endoglucanase, exoclucanase). Such systems have already been applied to *Lb. plantarum* [157,158], *Lb. gasseri* [159], *Lb. johnsonii* [159], and *L. lactis* [160,161]. Moreover, the production of other hydrolytic enzymes has also been reported. β-Galactosidases and lactases are important in the production of lactose-free dairy products, and they have been expressed in *Lb. plantarum* [162,163,164]. In addition, in *Lb. plantarum*, expression has been achieved of the chitosanase involved in cell-wall modifications [165] and the mannanase used for the hydrolysis of mannans [166,167].

Hyaluronic acid is used in the food and pharmaceutical industries. Hyaluronic acid is synthesized through hyaluronic acid synthase, by polymerization of uridine diphosphate (UDP)-glucuronic acid and UDP-N-acetylglucosamine, the two precursors of cell-wall components. Hyaluronic acid synthase from *Streptococcus equi* subsp. *zooepidemicus* was expressed in L. lactis and significantly enhanced hyaluronic acid production when co-expressed with hyaluronic acid synthase and UDP–glucose dehydrogenase [168], and similarly, when co-expressed with HA synthase, UDP–glucose dehydrogenase, and UDP–glucose pyrophosphorylase [169].

## 8. Conclusions

Genetic modification of LAB can result in improved strains with a wide spectrum of possible applications, including for therapies, the food industry, and metabolite production (e.g., as biocatalysts, cell factories). These applications differ in the acceptability and stringency of the required regulatory procedures. As biocatalysts, LAB can be used to produce lactic acid, pharmaceutical intermediates, nutraceuticals, and a range of chemicals. They represent promising cell factories for recombinant protein production and subsequent isolation, on account of their low tendency to form inclusion bodies and their lack of endotoxins. Improvements to LAB as cell factories by overexpression of the desired protein is more acceptable if it does not involve genetic engineering [170]. However, the GRAS or nonGMO status of these bacteria is not a necessity, but rather an added advantage, because of higher consumer acceptance [11]. Nevertheless, the minimal risk of dissemination of the microorganisms from closed systems affects the positive perception and acceptance of such LAB cell factories. 

The use of GM-LAB in food manufacturing is the most problematic, due to concerns related to the dissemination of modified stains, plasmids, and recombinant genes, and particularly due to low public acceptance. To enable the implementation of GM-LAB in the food industry possible, food-grade expression systems have been developed, which ensure the replacement of antibiotic-resistance markers with the use of alternative markers, and the use of homologous DNA. However, strain development for food use still relies on classical nonGMO methods [23,171]. 

Live LAB have been developed as biotherapeutics, especially for the treatment of gastrointestinal disorders [172,173,174]. NonGM-LAB have been readily used as probiotics, and fecal microbiota have already gained FDA acceptance for transplantation in the treatment of *Clostridium difficile* infection [175]. The development of engineered living biotherapeutics is expected to rise soon [176]. Several clinical trials with engineered LAB are in phase I or phase II [174], which define their necessity and their realistic therapeutic option. What is more, by 2030, microbiome therapeutics are expected to occupy close to 79% of the therapeutics segment, according to [177]. 

Compared to traditional methods for strain improvement, genome editing offers more precise procedures, and is consequently less likely to have unintended consequences. Recombineering and CRISPR-Cas9 techniques, in particular, will greatly facilitate targeted and trace-less genome modification. It is questionable whether a strain obtained via random mutagenesis (i.e., a method acceptable for regulatory bodies) is safer than a strain obtained via targeted and knowledge-based methods [113,178]. Therefore, all genome editing tools should not be treated in the same manner, but rather evaluated separately. However, the EU courts recently categorized new genome editing methods as subject to a 2001 directive, which thus defines them as GMO [179,180]. On the contrary, in the USA, a step toward application of next generation techniques has been made, with the use of Cas9-edited plants now allowed [181]. New legislation perspectives on the classification of new engineering techniques are required in the EU to allow the use of improved LAB strains that carry beneficial characteristics and novel functions. However, this remains a political, rather than a scientific, issue.

## Supplementary Materials

The following are available online at https://www.mdpi.com/2076-2607/8/2/297/s1. Table S1: Lactic acid bacteria ‘generally recognized as safe’ (GRAS) notices (adapted from GRAS Notices inventory; on 6 January 2020).

## References

[1] Stiles M.E., Holzapfel W.H. (1997). Lactic acid bacteria of foods and their current taxonomy. Int. J. Food Microbiol..

[2] Chowdhury M.Y., Li R., Kim J.H., Park M.E., Kim T.H., Pathinayake P., Weeratunga P., Song M.K., Son H.Y., Hong S.P. (2014). Mucosal vaccination with recombinant *Lactobacillus casei*-displayed CTA1-conjugated consensus matrix protein-2 (sM2) induces broad protection against divergent influenza subtypes in BALB/c mice. PLoS ONE.

[3] Van Braeckel-Budimir N., Haijema B.J., Leenhouts K. (2013). Bacterium-like particles for efficient immune stimulation of existing vaccines and new subunit vaccines in mucosal applications. Front. Immunol..

[4] William Reed NutraIngredients.com. https://www.nutraingredients.com/Article/2018/02/22/Consumer-acceptance-Novel-probiotics-are-beneficial-but-the-food-industry-is-its-own-worst-enemy-on-GM-technologies?utm_source=copyright&utm_medium=OnSite&utm_campaign=copyright.

[5] Ortiz-Velez L., Britton R. (2017). Genetic tools for the enhancement of probiotic properties. Microbiol. Spectr..

[6] Brodmann T., Endo A., Gueimonde M., Vinderola G., Kneifel W., de Vos W.M., Salminen S., Gomez-Gallego C. (2017). Safety of novel microbes for human consumption: Practical examples of assessment in the European Union. Front. Microbiol..

[7] Laulund S., Wind A., Derkx P.M.F., Zuliani V. (2017). Regulatory and safety requirements for food cultures. Microorganisms.

[8] O’Sullivan O., O’Callaghan J., Sangrador-Vegas A., McAuliffe O., Slattery L., Kaleta P., Callanan M., Fitzgerald G.F., Ross R.P., Beresford T. (2009). Comparative genomics of lactic acid bacteria reveals a niche-specific gene set. BMC Microbiol..

[9] Platteeuw C., van Alen-Boerrigter I., van Schalkwijk S., de Vos W.M. (1996). Food-grade cloning and expression system for *Lactococcus lactis*. Appl. Environ. Microbiol..

[10] Borner R.A., Kandasamy V., Axelsen A.M., Nielsen A.T., Bosma E.F. (2019). Genome editing of lactic acid bacteria: Opportunities for food, feed, pharma and Biotechnol. FEMS Microbiol. Lett..

[11] Hatti-Kaul R., Chen L., Dishisha T., Enshasy H.E. (2018). Lactic acid bacteria: From starter cultures to producers of chemicals. FEMS Microbiol. Lett..

[12] Tauer C., Heinl S., Egger E., Heiss S., Grabherr R. (2014). Tuning constitutive recombinant gene expression in *Lactobacillus plantarum*. Microb. Cell Fact..

[13] Ogaugwu C.E., Cheng Q., Fieck A., Hurwitz I., Durvasula R. (2017). Characterization of a *Lactococcus lactis* promoter for heterologous protein production. Biotechnol. Rep..

[14] de Vos W.M. (1999). Gene expression systems for lactic acid bacteria. Curr. Opin. Microbiol..

[15] Mays Z.J., Nair N.U. (2018). Synthetic biology in probiotic lactic acid bacteria: At the frontier of living therapeutics. Curr. Opin. Biotechnol..

[16] Dahmane N., Robert E., Deschamps J., Meylheuc T., Delorme C., Briandet R., Leblond-Bourget N., Guedon E., Payot S. (2018). Impact of cell surface molecules on conjugative transfer of the integrative and conjugative element ICESt3 of *Streptococcus thermophilus*. Appl. Environ. Microbiol..

[17] Chiang Y.N., Penades J.R., Chen J. (2019). Genetic transduction by phages and chromosomal islands: The new and noncanonical. PLoS Pathog..

[18] Baugher J.L., Durmaz E., Klaenhammer T.R. (2014). Spontaneously induced prophages in *Lactobacillus gasseri* contribute to horizontal gene transfer. Appl. Environ. Microbiol..

[19] Bron P.A., Marcelli B., Mulder J., van der Els S., Morawska L.P., Kuipers O.P., Kok J., Kleerebezem M. (2019). Renaissance of traditional DNA transfer strategies for improvement of industrial lactic acid bacteria. Curr. Opin. Biotechnol..

[20] Samson J.E., Moineau S. (2010). Characterization of *Lactococcus lactis* phage 949 and comparison with other lactococcal phages. Appl. Environ. Microbiol..

[21] Pujato S.A., Guglielmotti D.M., Martinez-Garcia M., Quiberoni A., Mojica F.J.M. (2017). *Leuconostoc mesenteroides* and *Leuconostoc pseudomesenteroides* bacteriophages: Genomics and cross-species host ranges. Int. J. Food Microbiol..

[22] Blokesch M. (2016). Natural competence for transformation. Curr. Biol..

[23] Derkx P.M., Janzen T., Sorensen K.I., Christensen J.E., Stuer-Lauridsen B., Johansen E. (2014). The art of strain improvement of industrial lactic acid bacteria without the use of recombinant DNA technology. Microb. Cell Fact..

[24] Chen J., Vestergaard M., Jensen T.G., Shen J., Dufva M., Solem C., Jensen P.R. (2017). Finding the needle in the haystack-the use of microfluidic droplet technology to identify vitamin-secreting lactic acid bacteria. mBio.

[25] Goodarzi A. (2016). UV—Induced mutagenesis in lactic acid bacteria. Int. J. Genet. Genom..

[26] Arihara K., Itoh M. (2000). UV-induced *Lactobacillus gasseri* mutants resisting sodium chloride and sodium nitrite for meat fermentation. Int. J. Food Microbiol..

[27] Almalki M.A. (2016). Production of medically important lactic acid by *Lactobacillus Pentosus*: A biological conversion method. Indian J. Sci. Technol..

[28] Mierau I., Kleerebezem M. (2005). 10 years of the nisin-controlled gene expression system (NICE) in *Lactococcus lactis*. Appl. Microbiol. Biotechnol..

[29] Nguyen T.T., Nguyen H.M., Geiger B., Mathiesen G., Eijsink V.G., Peterbauer C.K., Haltrich D., Nguyen T.H. (2015). Heterologous expression of a recombinant lactobacillal beta-galactosidase in *Lactobacillus plantarum*: Effect of different parameters on the sakacin P-based expression system. Microb. Cell Fact..

[30] Karlskas I.L., Maudal K., Axelsson L., Rud I., Eijsink V.G., Mathiesen G. (2014). Heterologous protein secretion in *Lactobacilli* with modified pSIP vectors. PLoS ONE.

[31] Sorvig E., Mathiesen G., Naterstad K., Eijsink V.G., Axelsson L. (2005). High-level, inducible gene expression in *Lactobacillus sakei* and *Lactobacillus plantarum* using versatile expression vectors. Microbiology.

[32] Walker D.C., Klaenhammer T.R. (1994). Isolation of a novel IS3 group insertion element and construction of an integration vector for *Lactobacillus* spp.. J. Bacteriol..

[33] Law J., Buist G., Haandrikman A., Kok J., Venema G., Leenhouts K. (1995). A system to generate chromosomal mutations in *Lactococcus lactis* which allows fast analysis of targeted genes. J. Bacteriol..

[34] Russell W.M., Klaenhammer T.R. (2001). Efficient system for directed integration into the *Lactobacillus acidophilus* and *Lactobacillus gasseri* chromosomes via homologous recombination. Appl. Environ. Microbiol..

[35] Neu T., Henrich B. (2003). New thermosensitive delivery vector and its use to enable nisin-controlled gene expression in *Lactobacillus gasseri*. Appl. Environ. Microbiol..

[36] Goh Y.J., Azcarate-Peril M.A., O’Flaherty S., Durmaz E., Valence F., Jardin J., Lortal S., Klaenhammer T.R. (2009). Development and application of a *upp*-based counterselective gene replacement system for the study of the S-layer protein SlpX of *Lactobacillus acidophilus* NCFM. Appl. Environ. Microbiol..

[37] Song L., Cui H., Tang L., Qiao X., Liu M., Jiang Y., Cui W., Li Y. (2014). Construction of *upp* deletion mutant strains of *Lactobacillus casei* and *Lactococcus lactis* based on counterselective system using temperature-sensitive plasmid. J. Microbiol. Meth..

[38] Lambert J.M., Bongers R.S., Kleerebezem M. (2007). Cre-lox-based system for multiple gene deletions and selectable-marker removal in *Lactobacillus plantarum*. Appl. Environ. Microbiol..

[39] Campo N., Daveran-Mingot M.L., Leenhouts K., Ritzenthaler P., Le Bourgeois P. (2002). Cre-loxP recombination system for large genome rearrangements in *Lactococcus lactis*. Appl. Environ. Microbiol..

[40] van Pijkeren J.P., Britton R.A. (2012). High efficiency recombineering in lactic acid bacteria. Nucleic Acids Res..

[41] Guo T., Xin Y., Zhang Y., Gu X., Kong J. (2019). A rapid and versatile tool for genomic engineering in *Lactococcus lactis*. Microb. Cell Fact..

[42] Oh J.H., van Pijkeren J.P. (2014). CRISPR-Cas9-assisted recombineering in *Lactobacillus reuteri*. Nucleic Acids Res..

[43] Hsu P.D., Lander E.S., Zhang F. (2014). Development and applications of CRISPR-Cas9 for genome engineering. Cell.

[44] Doudna J.A., Charpentier E. (2014). Genome editing. The new frontier of genome engineering with CRISPR-Cas9. Science.

[45] Selle K., Klaenhammer T.R., Barrangou R. (2015). CRISPR-based screening of genomic island excision events in bacteria. Proc. Natl. Acad. Sci. USA.

[46] van der Els S., James J.K., Kleerebezem M., Bron P.A. (2018). Development of a versatile Cas9-driven subpopulation-selection toolbox in *Lactococcus lactis*. Appl. Environ. Microbiol..

[47] Berlec A., Škrlec K., Kocjan J., Olenic M., Štrukelj B. (2018). Single plasmid systems for inducible dual protein expression and for CRISPR-Cas9/CRISPRi gene regulation in lactic acid bacterium *Lactococcus lactis*. Sci. Rep..

[48] Song X., Huang H., Xiong Z., Ai L., Yang S. (2017). CRISPR-Cas9(D10A) nickase-assisted genome editing in *Lactobacillus casei*. Appl. Environ. Microbiol..

[49] Qi L.S., Larson M.H., Gilbert L.A., Doudna J.A., Weissman J.S., Arkin A.P., Lim W.A. (2013). Repurposing CRISPR as an RNA-guided platform for sequence-specific control of gene expression. Cell.

[50] Mougiakos I., Mohanraju P., Bosma E.F., Vrouwe V., Finger Bou M., Naduthodi M.I.S., Gussak A., Brinkman R.B.L., van Kranenburg R., van der Oost J. (2017). Characterizing a thermostable Cas9 for bacterial genome editing and silencing. Nat. Commun..

[51] Nakade S., Yamamoto T., Sakuma T. (2017). Cas9, Cpf1 and C2c1/2/3-What’s next?. Bioengineered.

[52] Jiang Y., Qian F., Yang J., Liu Y., Dong F., Xu C., Sun B., Chen B., Xu X., Li Y. (2017). CRISPR-Cpf1 assisted genome editing of *Corynebacterium glutamicum*. Nat. Commun..

[53] Liang M., Li Z., Wang W., Liu J., Liu L., Zhu G., Karthik L., Wang M., Wang K.F., Wang Z. (2019). A CRISPR-Cas12a-derived biosensing platform for the highly sensitive detection of diverse small molecules. Nat. Commun..

[54] Shen W., Zhang J., Geng B., Qiu M., Hu M., Yang Q., Bao W., Xiao Y., Zheng Y., Peng W. (2019). Establishment and application of a CRISPR-Cas12a assisted genome-editing system in *Zymomonas mobilis*. Microb. Cell Fact..

[55] Kim Y.B., Komor A.C., Levy J.M., Packer M.S., Zhao K.T., Liu D.R. (2017). Increasing the genome-targeting scope and precision of base editing with engineered Cas9-cytidine deaminase fusions. Nat. Biotechnol..

[56] Gaudelli N.M., Komor A.C., Rees H.A., Packer M.S., Badran A.H., Bryson D.I., Liu D.R. (2017). Programmable base editing of A*T to G*C in genomic DNA without DNA cleavage. Nature.

[57] Eid A., Alshareef S., Mahfouz M.M. (2018). CRISPR base editors: Genome editing without double-stranded breaks. Biochem. J..

[58] Wang Y., Wang S., Chen W., Song L., Zhang Y., Shen Z., Yu F., Li M., Ji Q. (2018). CRISPR-Cas9 and CRISPR-assisted cytidine deaminase enable precise and efficient genome editing in *Klebsiella pneumoniae*. Appl. Environ. Microbiol..

[59] Zheng K., Wang Y., Li N., Jiang F.F., Wu C.X., Liu F., Chen H.C., Liu Z.F. (2018). Highly efficient base editing in bacteria using a Cas9-cytidine deaminase fusion. Commun. Biol..

[60] Wilson D.J. (1993). NIH guidelines for research involving recombinant DNA molecules. Account. Res..

[61] Lee P. (2010). Biocontainment strategies for live lactic acid bacteria vaccine vectors. Bioeng. Bugs.

[62] Steidler L., Neirynck S., Huyghebaert N., Snoeck V., Vermeire A., Goddeeris B., Cox E., Remon J.P., Remaut E. (2003). Biological containment of genetically modified *Lactococcus lactis* for intestinal delivery of human interleukin 10. Nat. Biotechnol..

[63] Bahey-El-Din M., Gahan C.G. (2010). *Lactococcus lactis*: From the dairy industry to antigen and therapeutic protein delivery. Discov. Med..

[64] Zhou H., Li X., Wang Z., Yin J., Tan H., Wang L., Qiao X., Jiang Y., Cui W., Liu M. (2018). Construction and characterization of thymidine auxotrophic (*ΔthyA*) recombinant *Lactobacillus casei* expressing bovine lactoferricin. BMC Vet. Res..

[65] Hanin A., Culligan E.P., Casey P.G., Bahey-El-Din M., Hill C., Gahan C.G. (2014). Two-tiered biological containment strategy for *Lactococcus lactis*-based vaccine or immunotherapy vectors. Hum. Vaccines Immunother..

[66] Callura J.M., Dwyer D.J., Isaacs F.J., Cantor C.R., Collins J.J. (2010). Tracking, tuning, and terminating microbial physiology using synthetic riboregulators. Proc. Natl. Acad. Sci. USA.

[67] Lee J.W., Chan C.T.Y., Slomovic S., Collins J.J. (2018). Next-generation biocontainment systems for engineered organisms. Nat. Chem. Biol..

[68] Gallagher R.R., Patel J.R., Interiano A.L., Rovner A.J., Isaacs F.J. (2015). Multilayered genetic safeguards limit growth of microorganisms to defined environments. Nucleic Acids Res..

[69] Mandell D.J., Lajoie M.J., Mee M.T., Takeuchi R., Kuznetsov G., Norville J.E., Gregg C.J., Stoddard B.L., Church G.M. (2015). Biocontainment of genetically modified organisms by synthetic protein design. Nature.

[70] Huang S., Lee A.J., Tsoi R., Wu F., Zhang Y., Leong K.W., You L. (2016). Coupling spatial segregation with synthetic circuits to control bacterial survival. Mol. Syst. Biol..

[71] Alvarez Y., Ponce-Alquicira E. (2018). Antibiotic Resistance in Lactic Acid Bacteria.

[72] Mendelson M., Matsoso M.P. (2015). The World Health Organization global action plan for antimicrobial resistance. S. Afr. Med. J..

[73] Markwart R., Willrich N., Haller S., Noll I., Koppe U., Werner G., Eckmanns T., Reuss A. (2019). The rise in vancomycin-resistant *Enterococcus faecium* in Germany: Data from the German Antimicrobial Resistance Surveillance (ARS). Antimicrob. Resist. Infect. Control..

[74] Faron M.L., Ledeboer N.A., Buchan B.W. (2016). Resistance mechanisms, epidemiology, and approaches to screening for vancomycin-resistant *Enterococcus* in the health care setting. J. Clin. Microbiol..

[75] Erginkaya Z., Turhan E.U., Tatli D. (2018). Determination of antibiotic resistance of lactic acid bacteria isolated from traditional Turkish fermented dairy products. Iran. J. Vet. Res..

[76] Gad G.F., Abdel-Hamid A.M., Farag Z.S. (2014). Antibiotic resistance in lactic acid bacteria isolated from some pharmaceutical and dairy products. Braz. J. Microbiol..

[77] Guo H., Pan L., Li L., Lu J., Kwok L., Menghe B., Zhang H., Zhang W. (2017). Characterization of antibiotic resistance genes from *Lactobacillus* isolated from traditional dairy products. J. Food Sci..

[78] Liu C., Zhang Z.Y., Dong K., Yuan J.P., Guo X.K. (2009). Antibiotic resistance of probiotic strains of lactic acid bacteria isolated from marketed foods and drugs. Biomed. Environ. Sci..

[79] Doucet-Populaire F., Trieu-Cuot P., Andremont A., Courvalin P. (1992). Conjugal transfer of plasmid DNA from *Enterococcus faecalis* to *Escherichia coli* in digestive tracts of gnotobiotic mice. Antimicrob. Agents Chemother..

[80] Bolotin A., Quinquis B., Sorokin A., Ehrlich D.S. (2004). Recent genetic transfer between *Lactococcus lactis* and *enterobacteria*. J. Bacteriol..

[81] Mater D.D.G., Langella P., Corthier G., Flores M.J. (2008). A probiotic *Lactobacillus* strain can acquire vancomycin resistance during digestive transit in mice. J. Mol. Microb. Biotechnol..

[82] International Organization for Standardization Milk and Milk Products: Determination of the Minimal Inhibitory Concentration (MIC) of Antibiotics Applicable to *Bifidobacteria* and *Non-Enterococcal* Lactic Acid Bacteria. https://www.iso.org/standard/46434.html.

[83] Clementi F., Aquilanti L. (2011). Recent investigations and updated criteria for the assessment of antibiotic resistance in food lactic acid bacteria. Anaerobe.

[84] De R. (2019). Metagenomics: Aid to combat antimicrobial resistance in diarrhea. Gut Pathog..

[85] Schloss P.D., Handelsman J. (2003). Biotechnological prospects from metagenomics. Curr. Opin. Biotechnol..

[86] European Food Safety A. (2008). Technical guidance - Update of the criteria used in the assessment of bacterial resistance to antibiotics of human or veterinary importance. EFSA J..

[87] Cox G., Wright G.D. (2013). Intrinsic antibiotic resistance: Mechanisms, origins, challenges and solutions. Int. J. Med. Microbiol..

[88] Mignon C., Sodoyer R., Werle B. (2015). Antibiotic-free selection in biotherapeutics: Now and forever. Pathogens.

[89] He S., Gong F., Zhang D., Guo Y. (2012). Food-grade selection markers in lactic acid bacteria. TAF Prev. Med. Bull..

[90] Hughes B.F., McKay L.L. (1992). Deriving phage-insensitive *Lactococci* using a food-grade vector encoding phage and nisin resistance. J. Dairy Sci..

[91] von Wright A., Wessels S., Tynkkynen S., Saarela M. (1990). Isolation of a replication region of a large *lactococcal* plasmid and use in cloning of a nisin resistance determinant. Appl. Environ. Microbiol..

[92] Takala T., Saris P. (2002). A food-grade cloning vector for lactic acid bacteria based on the nisin immunity gene nisI. Appl. Microbiol. Biotechnol..

[93] Leelawatcharamas V., Chia L.G., Charoenchai P., Kunajakr N., Liu C.-Q., Dunn N.W. (1997). Plasmid-encoded copper resistance in *Lactococcus lactis*. Biotechnol. Lett..

[94] Liu C.Q., Su P., Khunajakr N., Deng Y.M., Sumual S., Kim W.S., Tandianus J.E., Dunn N.W. (2005). Development of food-grade cloning and expression vectors for *Lactococcus lactis*. J. Appl. Microbiol..

[95] Wong W.Y., Su P., Allison G.E., Liu C.-Q., Dunn N.W. (2003). A potential food-grade cloning vector for *Streptococcus thermophilus* that uses cadmium resistance as the selectable marker. Appl. Environ. Microbiol..

[96] El Demerdash H.A.M., Heller K.J., Geis A. (2003). Application of the shsp gene, encoding a small heat shock protein, as a food-grade selection marker for lactic acid bacteria. Appl. Environ. Microbiol..

[97] Sørensen K.I., Larsen R., Kibenich A., Junge M.P., Johansen E. (2000). A food-grade cloning system for industrial strains of *Lactococcus lactis*. Appl. Environ. Microbiol..

[98] Bron P.A., Benchimol M.G., Lambert J., Palumbo E., Deghorain M., Delcour J., De Vos W.M., Kleerebezem M., Hols P. (2002). Use of the alr gene as a food-grade selection marker in lactic acid bacteria. Appl. Environ. Microbiol..

[99] Takala T., Saris P., Tynkkynen S. (2003). Food-grade host/vector expression system for *Lactobacillus casei* based on complementation of plasmid-associated phospho-β-galactosidase gene lacG. Appl. Microbiol. Biotechnol..

[100] Boucher I., Parrot M., Gaudreau H., Champagne C.P., Vadeboncoeur C., Moineau S. (2002). Novel food-grade plasmid vector based on melibiose fermentation for the genetic engineering of *Lactococcus lactis*. Appl. Environ. Microbiol..

[101] Labrie S., Bart C., Vadeboncoeur C., Moineau S. (2005). Use of an α-galactosidase gene as a food-grade selection marker for *Streptococcus thermophilus*. J. Dairy Sci..

[102] Jeong D.-W., Lee J.-H., Kim K.H., Lee H.J. (2006). A food-grade expression/secretion vector for *Lactococcus lactis* that uses an α-galactosidase gene as a selection marker. Food Microbiol..

[103] Peubez I., Chaudet N., Mignon C., Hild G., Husson S., Courtois V., De Luca K., Speck D., Sodoyer R. (2010). Antibiotic-free selection in *E. coli*: New considerations for optimal design and improved production. Microb. Cell Fact..

[104] Shao Y., Harrison E.M., Bi D., Tai C., He X., Ou H.-Y., Rajakumar K., Deng Z. (2011). TADB: A web-based resource for type 2 toxin-antitoxin loci in bacteria and archaea. Nucleic Acids Res..

[105] Diago-Navarro E., Hernandez-Arriaga A.M., López-Villarejo J., Muñoz-Gómez A.J., Kamphuis M.B., Boelens R., Lemonnier M., Díaz-Orejas R. (2010). parD toxin–antitoxin system of plasmid R1–basic contributions, biotechnological applications and relationships with closely-related toxin–antitoxin systems. FEBS J..

[106] Kawano M. (2012). Divergently overlapping cis-encoded antisense RNA regulating toxin-antitoxin systems from *E. coli*: Hok/sok, ldr/rdl, symE/symR. RNA Biol..

[107] Weaver K.E. (2012). The par toxin-antitoxin system from *Enterococcus faecalis* plasmid pAD1 and its chromosomal homologs. RNA Biol..

[108] Weaver K.E. (2015). The Type I toxin-antitoxin par locus from *Enterococcus faecalis* plasmid pAD1: RNA regulation by both cis- and trans-acting elements. Plasmid.

[109] Mairhofer J., Pfaffenzeller I., Merz D., Grabherr R. (2008). A novel antibiotic free plasmid selection system: Advances in safe and efficient DNA therapy. Biotechnol. J..

[110] Pfaffenzeller I., Mairhofer J., Striedner G., Bayer K., Grabherr R. (2006). Using ColE1-derived RNA I for suppression of a bacterially encoded gene: Implication for a novel plasmid addiction system. Biotechnol. J..

[111] Stenler S., Blomberg P., Smith C.I.E. (2014). Safety and efficacy of DNA vaccines: Plasmids vs. minicircles. Hum. Vaccin. Immunother..

[112] Douglas G.L., Goh Y.J., Klaenhammer T.R. (2011). Integrative food grade expression system for lactic acid bacteria. Methods Mol. Biol..

[113] Johansen E. (2017). Future access and improvement of industrial lactic acid bacteria cultures. Microb. Cell Fact..

[114] Phumkhachorn P., Rattanachaikunsopon P. (2016). A broad host range food-grade cloning vector for lactic acid bacteria. Biologia.

[115] Tagliavia M., Nicosia A. (2019). Advanced strategies for food-grade protein production: A new *E. coli*/lactic acid bacteria shuttle vector for improved cloning and food-grade expression. Microorganisms.

[116] Mathipa M.G., Thantsha M.S. (2017). Probiotic engineering: Towards development of robust probiotic strains with enhanced functional properties and for targeted control of enteric pathogens. Gut Pathog..

[117] Sybesma W., Hugenholtz J., Vos W.M.D., Smid E.J. (2006). Safe use of genetically modified lactic acid bacteria in food. Bridging the gap between consumers, green groups, and industry. Electron. J. Biotechnol..

[118] Salminen M.K., Tynkkynen S., Rautelin H., Saxelin M., Vaara M., Ruutu P., Sarna S., Valtonen V., Järvinen A. (2002). *Lactobacillus* bacteremia during a rapid increase in probiotic use of *Lactobacillus rhamnosus* GG in Finland. Clin. Infect. Dis..

[119] Zadravec P., Štrukelj B., Berlec A. (2015). Heterologous surface display on lactic acid bacteria: Non-GMO alternative?. Bioengineered.

[120] Hu S., Kong J., Kong W., Guo T., Ji M. (2010). Characterization of a novel LysM domain from *Lactobacillus fermentum* bacteriophage endolysin and its use as an anchor to display heterologous proteins on the surfaces of lactic acid bacteria. Appl. Environ. Microbiol..

[121] Hu S., Kong J., Sun Z., Han L., Kong W., Yang P. (2011). Heterologous protein display on the cell surface of lactic acid bacteria mediated by the s-layer protein. Microb. Cell Fact..

[122] Plavec T.V., Štrukelj B., Berlec A. (2019). Screening for new surface anchoring domains for *Lactococcus lactis*. Front. Microbiol..

[123] Plavec T.V., Berlec A. (2019). Surface anchoring on *Lactococcus lactis* by covalent isopeptide bond. Acta Chim. Slov..

[124] Mao R., Wu D., Wang Y. (2016). Surface display on lactic acid bacteria without genetic modification: Strategies and applications. Appl. Microbiol. Biotechnol..

[125] Song A.A., In L.L.A., Lim S.H.E., Rahim R.A. (2017). A review on *Lactococcus lactis*: From food to factory. Microb. Cell Fact..

[126] Berlec A., Štrukelj B. (2009). Novel applications of recombinant lactic acid bacteria in therapy and in metabolic engineering. Recent Pat. Biotechnol..

[127] Hugenholtz J., Sybesma W., Nierop Groot M., Wisselink W., Ladero V., Burgess K., van Sinderen D., Piard J.-C., Eggink G., Smid E.J. (2002). Metabolic engineering of lactic acid bacteria for the production of nutraceuticals. Lactic Acid Bacteria Genet. Metab. Appl..

[128] Liu J., Chan S.H.J., Chen J., Solem C., Jensen P.R. (2019). Systems Biology-A guide for understanding and developing improved strains of lactic acid bacteria. Front. Microbiol..

[129] Papagianni M. (2012). Metabolic engineering of lactic acid bacteria for the production of industrially important cpmpounds. Comput. Struct. Biotechnol..

[130] Kleerebezem M., Hugenholtz J. (2003). Metabolic pathway engineering in lactic acid bacteria. Curr. Opin. Biotechnol..

[131] Castro-Aguirre E., Iniguez-Franco F., Samsudin H., Fang X., Auras R. (2016). Poly(lactic acid)-mass production, processing, industrial applications, and end of life. Adv. Drug Deliv. Rev..

[132] Mack D.R. (2004). D(-)-lactic acid-producing probiotics, D(-)-lactic acidosis and infants. Can. J. Gastroenterol..

[133] Yu L., Pei X., Lei T., Wang Y., Feng Y. (2008). Genome shuffling enhanced L-lactic acid production by improving glucose tolerance of *Lactobacillus rhamnosus*. J. Biotechnol..

[134] Yi X., Zhang P., Sun J., Tu Y., Gao Q., Zhang J., Bao J. (2016). Engineering wild-type robust *Pediococcus acidilactici* strain for high titer L- and D-lactic acid production from corn stover feedstock. J. Biotechnol..

[135] Okano K., Hama S., Kihara M., Noda H., Tanaka T., Kondo A. (2017). Production of optically pure D-lactic acid from brown rice using metabolically engineered *Lactobacillus plantarum*. Appl. Microbiol. Biotechnol..

[136] Okano K., Zhang Q., Shinkawa S., Yoshida S., Tanaka T., Fukuda H., Kondo A. (2009). Efficient production of optically pure D-lactic acid from raw corn starch by using a genetically modified L-lactate dehydrogenase gene-deficient and alpha-amylase-secreting *Lactobacillus plantarum* strain. Appl. Environ. Microbiol..

[137] Kuo Y.C., Yuan S.F., Wang C.A., Huang Y.J., Guo G.L., Hwang W.S. (2015). Production of optically pure L-lactic acid from lignocellulosic hydrolysate by using a newly isolated and D-lactate dehydrogenase gene-deficient *Lactobacillus paracasei* strain. Bioresour. Technol..

[138] Kyla-Nikkila K., Hujanen M., Leisola M., Palva A. (2000). Metabolic engineering of *Lactobacillus helveticus* CNRZ32 for production of pure L-(+)-lactic acid. Appl. Environ. Microbiol..

[139] Liu J., Dantoft S.H., Würtz A., Jensen P.R., Solem C. (2016). A novel cell factory for efficient production of ethanol from dairy waste. Biotechnol. Biofuels.

[140] Florou-Paneri P., Christaki E., Bonos E. (2012). Lactic Acid Bacteria as Source of Functional Ingredients.

[141] Linares D.M., Gómez C., Renes E., Fresno J.M., Tornadijo M.E., Ross R.P., Stanton C. (2017). Lactic acid bacteria and *Bifidobacteria* with potential to design natural biofunctional health-promoting dairy foods. Front. Microbiol..

[142] Zhang J., Caiyin Q., Feng W., Zhao X., Qiao B., Zhao G., Qiao J. (2016). Enhance nisin yield via improving acid-tolerant capability of *Lactococcus lactis* F44. Sci. Rep..

[143] Fu Y., Mu D., Qiao W., Zhu D., Wang X., Liu F., Xu H., Saris P., Kuipers O.P., Qiao M. (2018). Co-expression of nisin Z and leucocin C as a basis for effective protection against *Listeria monocytogenes* in pasteurized milk. Front. Microbiol..

[144] Burgess C., O’Connell-Motherway M., Sybesma W., Hugenholtz J., van Sinderen D. (2004). Riboflavin production in *Lactococcus lactis*: Potential for in situ production of vitamin-enriched foods. Appl. Environ. Microbiol..

[145] LeBlanc J.G., Burgess C., Sesma F., de Giori G.S., van Sinderen D. (2005). Ingestion of milk fermented by genetically modified *Lactococcus lactis* improves the riboflavin status of deficient rats. J. Dairy Sci..

[146] Wegkamp A., van Oorschot W., de Vos W.M., Smid E.J. (2007). Characterization of the role of para-aminobenzoic acid biosynthesis in folate production by *Lactococcus lactis*. Appl. Environ. Microbiol..

[147] Wegkamp A., Starrenburg M., de Vos W.M., Hugenholtz J., Sybesma W. (2004). Transformation of folate-consuming *Lactobacillus gasseri* into a folate producer. Appl. Environ. Microbiol..

[148] Santos F., Wegkamp A., de Vos W.M., Smid E.J., Hugenholtz J. (2008). High-level folate production in fermented foods by the B12 producer *Lactobacillus reuteri* JCM1112. Appl. Environ. Microbiol..

[149] De Boeck R., Sarmiento-Rubiano L.A., Nadal I., Monedero V., Perez-Martinez G., Yebra M.J. (2010). Sorbitol production from lactose by engineered *Lactobacillus casei* deficient in sorbitol transport system and mannitol-1-phosphate dehydrogenase. Appl. Microbiol. Biotechnol..

[150] Ladero V., Ramos A., Wiersma A., Goffin P., Schanck A., Kleerebezem M., Hugenholtz J., Smid E.J., Hols P. (2007). High-level production of the low-calorie sugar sorbitol by *Lactobacillus plantarum* through metabolic engineering. Appl. Environ. Microbiol..

[151] Gaspar P., Neves A.R., Gasson M.J., Shearman C.A., Santos H. (2011). High yields of 2,3-butanediol and mannitol in *Lactococcus lactis* through engineering of NAD(+) cofactor recycling. Appl. Environ. Microbiol..

[152] Gaspar P., Carvalho A.L., Vinga S., Santos H., Neves A.R. (2013). From physiology to systems metabolic engineering for the production of biochemicals by lactic acid bacteria. Biotechnol. Adv..

[153] Mazzoli R., Bosco F., Mizrahi I., Bayer E.A., Pessione E. (2014). Towards lactic acid bacteria-based biorefineries. Biotechnol. Adv..

[154] Narita J., Ishida S., Okano K., Kimura S., Fukuda H., Kondo A. (2006). Improvement of protein production in lactic acid bacteria using 5′-untranslated leader sequence of slpA from *Lactobacillus acidophilus*. Appl. Microbiol. Biotechnol..

[155] Narita J., Nakahara S., Fukuda H., Kondo A. (2004). Efficient production of L-(+)-lactic acid from raw starch by *Streptococcus bovis* 148. J. Biosci. Bioeng..

[156] Okano K., Kimura S., Narita J., Fukuda H., Kondo A. (2007). Improvement in lactic acid production from starch using α-amylase-secreting *Lactococcus lactis* cells adapted to maltose or starch. Appl. Microbiol. Biotechnol..

[157] Okano K., Zhang Q., Yoshida S., Tanaka T., Ogino C., Fukuda H., Kondo A. (2010). d-lactic acid production from cellooligosaccharides and β-glucan using l-LDH gene-deficient and endoglucanase-secreting *Lactobacillus plantarum*. Appl. Microbiol. Biotechnol..

[158] Moraïs S., Shterzer N., Grinberg I.R., Mathiesen G., Eijsink V.G.H., Axelsson L., Lamed R., Bayer E.A., Mizrahi I. (2013). Establishment of a simple *Lactobacillus plantarum* cell consortium for cellulase-xylanase synergistic interactions. Appl. Environ. Microbiol..

[159] Cho J.S., Choi Y.J., Chung D.K. (2000). Expression of *Clostridium thermocellum* endoglucanase gene in *Lactobacillus gasseri* and *Lactobacillus johnsonii* and characterization of the genetically modified probiotic *Lactobacilli*. Curr. Microbiol..

[160] Raha A.R., Chang L.Y., Sipat A., Yusoff K., Haryanti T. (2006). Expression of a thermostable xylanase gene from *Bacillus coagulans* ST-6 in *Lactococcus lactis*. Lett. Appl. Microbiol..

[161] Ozkose E., Akyol I., Kar B., Comlekcioglu U., Ekinci M.S. (2009). Expression of fungal cellulase gene in *Lactococcus lactis* to construct novel recombinant silage inoculants. Folia Microbiol..

[162] Nguyen T.H., Splechtna B., Yamabhai M., Haltrich D., Peterbauer C. (2007). Cloning and expression of the beta-galactosidase genes from *Lactobacillus reuteri* in *Escherichia coli*. J. Biotechnol..

[163] Nguyen T.T., Nguyen H.A., Arreola S.L., Mlynek G., Djinovic-Carugo K., Mathiesen G., Nguyen T.H., Haltrich D. (2012). Homodimeric beta-galactosidase from *Lactobacillus delbrueckii* subsp. *bulgaricus* DSM 20081: Expression in *Lactobacillus plantarum* and biochemical characterization. J. Agric. Food Chem..

[164] Halbmayr E., Mathiesen G., Nguyen T.H., Maischberger T., Peterbauer C.K., Eijsink V.G., Haltrich D. (2008). High-level expression of recombinant beta-galactosidases in *Lactobacillus plantarum* and *Lactobacillus sakei* using a sakacin P-based expression system. J. Agric. Food Chem..

[165] Nguyen H.M., Mathiesen G., Stelzer E.M., Pham M.L., Kuczkowska K., Mackenzie A., Agger J.W., Eijsink V.G., Yamabhai M., Peterbauer C.K. (2016). Display of a beta-mannanase and a chitosanase on the cell surface of *Lactobacillus plantarum* towards the development of whole-cell biocatalysts. Microb. Cell Fact..

[166] Yang P., Li Y., Wang Y., Meng K., Luo H., Yuan T., Bai Y., Zhan Z., Yao B. (2009). A novel beta-mannanase with high specific activity from *Bacillus circulans* CGMCC1554: Gene cloning, expression and enzymatic characterization. Appl. Biochem. Biotechnol..

[167] Sak-Ubol S., Namvijitr P., Pechsrichuang P., Haltrich D., Nguyen T.H., Mathiesen G., Eijsink V.G., Yamabhai M. (2016). Secretory production of a beta-mannanase and a chitosanase using a *Lactobacillus plantarum* expression system. Microb. Cell Fact..

[168] Chien L.-J., Lee C.-K. (2007). Hyaluronic acid production by recombinant *Lactococcus lactis*. Appl. Microbiol. Biotechnol..

[169] Prasad S.B., Jayaraman G., Ramachandran K.B. (2010). Hyaluronic acid production is enhanced by the additional co-expression of UDP-glucose pyrophosphorylase in *Lactococcus lactis*. Appl. Microbiol. Biotechnol..

[170] Thakur K., Tomar S.K., De S. (2016). Lactic acid bacteria as a cell factory for riboflavin production. Microb. Biotechnol..

[171] Eric J. (2018). Use of Natural selection and evolution to develop new starter cultures for fermented foods. Annu. Rev. Food Sci. Technol..

[172] Carvalho R.D., do Carmo F.L.R., de Oliveira Junior A., Langella P., Chatel J.-M., Bermúdez-Humarán L.G., Azevedo V., de Azevedo M.S. (2017). Use of wild type or recombinant lactic acid bacteria as an alternative treatment for gastrointestinal inflammatory diseases: A focus on inflammatory bowel diseases and mucositis. Front. Microbiol..

[173] de Moreno de LeBlanc A., Del Carmen S., Chatel J.-M., Miyoshi A., Azevedo V., Langella P., Bermúdez-Humarán L.G., LeBlanc J.G. (2015). Current review of genetically modified lactic acid bacteria for the prevention and treatment of colitis using murine models. Gastroent. Res. Pract..

[174] Plavec T.V., Berlec A. (2019). Engineering of lactic acid bacteria for delivery of therapeutic proteins and peptides. Appl. Microbiol. Biotechnol..

[175] Food and Drug Administration (2013). Enforcement Policy Regarding Investigational New Drug Requirements for Use of Fecal Microbiota for Transplantation to Treat *Clostridium difficile* Infection Not Responsive to Standard Therapies. Center for Biologics Evaluation and Research. https://www.fda.gov/regulatory-information/search-fda-guidance-documents/enforcement-policy-regarding-investigational-new-drug-requirements-use-fecal-microbiota.

[176] Bron P.A., Kleerebezem M. (2018). Lactic acid bacteria for delivery of endogenous or engineered therapeutic molecules. Front. Microbiol..

[177] Research and Markets Microbiome Therapeutics and Diagnostics Market (2nd Edition), 2017–2030. https://www.researchandmarkets.com/reports/4377904/microbiome-therapeutics-and-diagnostics-market.

[178] Custers R. (2017). The regulatory status of gene-edited agricultural products in the EU and beyond. Emerg. Top. Life Sci..

[179] Callaway E. (2018). CRISPR plants now subject to tough GM laws in European Union. Nature.

[180] Shew A.M., Nalley L.L., Snell H.A., Nayga R.M., Dixon B.L. (2018). CRISPR versus GMOs: Public acceptance and valuation. Glob. Food Secur..

[181] Wang T., Zhang H., Zhu H. (2019). CRISPR technology is revolutionizing the improvement of tomato and other fruit crops. Hortic. Res..

