# Supplementary material for: Safety Aspects of Genetically Modified Lactic Acid Bacteria
Source: Microorganisms. 2020 Feb 21;8(2):297. doi: 10.3390/microorganisms8020297 (PMC7074969; doi:10.3390/microorganisms8020297)
Supplement: Supplementary file 1 [file microorganisms-08-00297-s001.pdf]

Supplementary Table S1: Lactic acid bacteria 'generally recognized as safe' (GRAS) notices (adapted from GRAS Notices inventory; on Jan 6, 2020).

Substance	Intended use	GRN No.	Date of closure
Lactobacillus plantarum ECGC 13110402	Ingredient in foods generally, excluding infant formulae and foods that would require additional regulatory review by USDA, at levels up to 1 x10^{10} CFU/serving	847	Sept 30, 2019
Lactobacillus paracasei strain F19	Probiotic microorganism in conventional foods, excluding USDA-regulated foods, at levels intended to provide at a minimum of 10^9 CFU/serving	840	Aug 27, 2019
Lactobacillus paracasei subsp. *paracasei* strain F-19e	Ingredient in powdered-milk-based nonexempt infant formulae at levels intended to provide a daily intake of 10^9 CFU of the strain	810	Apr 5, 2019
Lactobacillus curvatus DSM 18775	To suppress the growth of *Listeria monocytogenes* in ready-to-eat cooked meat and poultry products	760	Jan 29, 2019
Lactobacillus helveticus R0052, *Bifidobacterium longum* subsp. *infantis* R0033, and *Bifidobacterium bifidum* R0071	Both individually and in combination, as an ingredient of powdered infant formulae at 5 x10^7 CFU/g of powder in formulae with hydration rates of 12.5 to 13/5 g/100 mL	758	Aug 20, 2018
Lactobacillus casei subsp. *paracasei* Lpc-37	Ingredient in yogurt and other dairy products, soy products, beverages, chewing gum, and confectionary snacks	736	Apr 11, 2018
Lactobacillus plantarum Lp-115	Ingredient in yogurt and other dairy products; soy products; beverages; chewing gum, confectionary snacks and other foods at levels not more than 5 x10^{11} CFU/serving	722	Feb 16, 2018
Lactobacillus plantarum strain 299v	Probiotic microorganism in conventional foods at a use level of up to 10^{11} CFU/serving	685	Oct 31, 2017
Lactobacillus fermentum CECT5716	In powdered-milk-based infant formulae at 10^7 CFU/g of powdered formula	531	Mar 20, 2015
Lactobacillus acidophilus La-14	Ingredient in certain food categories at a level of 10^9 CFU/250 g serving of food at the time of consumption	502	Aug 19, 2014
Lactobacillus reuteri strain NCIMB 30242	Ingredient in beverages and beverage bases, breakfast cereals, cheeses, dairy product analogs, fats and oils, frozen dairy desserts, grain products and pasta, milk products, processed fruit and fruit juices, and sugar substitutes at levels ranging from 3.3 x10^8 to 10^{10} CFU/serving	440	Feb 12, 2013
Lactobacillus casei strain Shirota	Ingredient in fermented dairy products at a maximum level of 4 x10^8 CFU/mL	429	Dec 10, 2012
Lactobacillus reuteri strain DSM 17938	Ingredient in powdered-whey-based term infant formulae at a minimum level of 10^6 CFU/g, but not higher than 10^8 CFU/g of powdered formula, produced in accordance with current good manufacturing practices	410	Mar 26, 2012
Lactobacillus acidophilus NCFM	Ingredient in certain dairy products, functional beverages, nutritional powders, juices, bars, ready-to-eat breakfast cereals, chewing gum, and confectionery, at levels not to exceed current good manufacturing practice in accordance with 21 CFR 184.1(b)	357	Apr 19, 2011
Lactobacillus rhamnosus strain HN001	Ingredient in various foods, including certain beverages and beverage bases (excluding soft drinks); cheeses; milk drinks; milk products; meal replacements; energy bars; ready-to-eat cereals; fruit juices, nectars, ades, and drinks; confectionery; chewing gum, and hard candies at a level to provide up to 10^9 CFU/standard serving	288	Nov 1, 2009
Lactobacillus rhamnosus strain HN001 produced in a milk-based medium	Ingredient in milk-based powdered term infant formulae that is intended for consumption from the time of birth, as well as	281	Aug 31, 2009

	in milk-based powdered follow-on formulae, at a level of 10^8 CFU/g of the formula powder		
Lactobacillus reuteri strain DSM 17938	Ingredient in processed cheeses, yogurt, ice cream, fruit juices, fruit drinks, processed vegetables, processed vegetable drinks, beverage bases, energy bars, energy drinks, and chewing gum at a level up to 10^9 CFU/serving, and in a drinking straw at a level of 10^9 CFU/straw	254	Nov 18, 2008
Lactobacillus casei subsp. *rhamnosus* strain GG	Ingredient in term infant formulae, at levels not to exceed 10^8 CFU/g of powdered formula	231	May 29, 2008
Lactobacillus acidophilus, *Lactobacillus lactis*, and *Pediococcus acidilactici*	Antimicrobial to control pathogenic bacteria in meat and poultry products	171	Dec 7, 2005
Streptococcus salivarius M18	Ingredient in baby, infant, and toddler foods (excluding infant formulae); baked goods and baking mixes; beverage and beverage bases; breakfast cereals; cheeses; chewing gum; dairy product analogs; frozen dairy desserts and mixes; gelatins, puddings, and fillings; grain products and pastas; hard candy; milk, whole and skimmed; milk products; nuts and nut products; processed fruit and fruit juices; soft candy; sweet sauces, toppings, and syrups at a level of 20 mg/serving, providing a minimum of 1 $x10^9$ CFU/serving	807	Jun 6, 2019
Streptococcus salivarius K12	Ingredient in baby, infant, and toddler food (excluding infant formulae), baked goods and baking mixes, beverage and beverage bases, breakfast cereals, cheeses, chewing gum, dairy product analogs, frozen dairy desserts and mixes, gelatins, puddings and fillings, grain products and pasta, hard candy, milk, whole and skimmed; milk products, nuts and nut products, processed fruit and fruit juices, soft candy, sweet sauces, toppings and syrups, and medical foods, at levels up to 20 mg/serving (providing a minimum of 2 $x10^9$ CFU/serving)	591	Jan 25, 2016
Carnobacterium maltaromaticum strain CB1 (viable and heat-treated)	Inhibitor of *Listeria monocytogenes* in a variety of foods including meat and poultry, as follows: (1) viable *C. maltaromaticum* CB1 in ready-to-eat meat products; meat, poultry, and fish products; frozen meals; processed fruit salads and vegetable salads; sauces; and, soft cheese and cheese spread-type products; and (2) heat-treated *C. maltaromaticum* CB1 as an inhibitor of *L. monocytogenes* in ready-to-eat meat products, meat, poultry, fish, and mixtures; fruit and vegetables; soups and stews; sauces and dressings; cheese and cheese spread-type products	305	Apr 19, 2010
Carnobacterium maltaromaticum strain CB1	Inhibitor of the growth of *Listeria monocytogenes* in ready-to-eat meat products	159	May 27, 2005
Bifidobacterium lactis strain Bb12 and *Streptococcus thermophilus* strain Th4	Ingredients in milk-based infant formulae that is intended for consumption by infants four months and older, at levels not to exceed good manufacturing practice	49	Mar 19, 2002
Cultured [dairy sources, sugars, wheat, malt, and fruit- and vegetable-based sources] fermented by [*Streptococcus thermophilus*, *Bacillus coagulans*, *Lactobacillus acidophilus*, *Lactobacillus paracasei* subsp. *paracasei*, *Lactobacillus plantarum*, *Lactobacillus sakei*, *Lactobacillus bulgaricus* and	Antimicrobial agents in a variety of food categories typically at levels of 0.1% to 4.5%, including meat and poultry, but excepting infant formulae and infant foods	378	Mar 26, 2012

Proprionibacterium freudenreichii subsp. *shermanii* or mixtures of these strains]			
Corn, cane, or beet sugar cultured with *Lactobacillus paracasei* subsp. *paracasei*, *Bacillus coagulans* LA-1, or *Propionibacterium freudenreichii* subsp. *shermanii*, or mixtures of these microorganisms	Antimicrobial agent in meat and poultry products at a level of 4.8%	240	Oct 24, 2008
Sodium formate	Support of the growth of *Streptococcus*, *Lactobacillus*, and *Leuconostoc* species in fermented dairy and soy products at levels not to exceed 20 parts per million	668	Mar 9, 2017
